# Potential Application of Recombinant Snake Prothrombin Activator Ecarin in Blood Diagnostics

**DOI:** 10.3390/biom12111704

**Published:** 2022-11-17

**Authors:** Kong-Nan Zhao, Paul Masci, Goce Dimeski, Lambro Johnson, Michael Grant, John de Jersey, Martin F. Lavin

**Affiliations:** 1 Australian Institute of Biotechnology and Nanotechnology, St Lucia Campus, The University of Queensland, Brisbane, QLD 4072, Australia; 2 Centre for Kidney Disease Research-Venomics Research, School of Medicine, The University of Queensland, Brisbane, QLD 4072, Australia; 3 Chemical Pathology, Princess Alexandra Hospital, Woolloongabba, Brisbane, QLD 4102, Australia; 4 School of Chemistry and Molecular Biosciences, St Lucia Campus, The University of Queensland, Brisbane, QLD 4072, Australia; 5 Q-Sera Pty Ltd., Level 9, 31 Queen St, Melbourne, VIC 3000, Australia; 6 Centre for Clinical Research, RBWH Campus, The University of Queensland, Brisbane, QLD 4029, Australia

**Keywords:** recombinant ecarin, blood clotting, clotting of anticoagulated blood, high quality serum

## Abstract

We describe here the purification and cloning of a codon-optimized form of the snake prothrombin activator ecarin from the saw scaled viper (*Echis carinatus*) expressed in mammalian cells. Expression of recombinant ecarin (rEcarin) was carried out in human embryonic kidney cells (HEK) cells under conditions for the development and performance of a novel and scalable recombinant snake ecarin to industry standards. Clotting performance of the rEcarin was established in recalcified citrated whole blood, plasma, and fresh whole blood and found to be comparable to native ecarin (N-Ecarin). Furthermore, hemolysis was observed with N-Ecarin at relatively high doses in both recalcified citrated and fresh whole blood, while clotting was not observed with rEcarin, providing an important advantage for the recombinant form. In addition, rEcarin effectively clotted both recalcified citrated whole blood and fresh whole blood containing different anticoagulants including heparin, warfarin, dabigatran, Fondaparinux, rivaroxaban and apixaban, forming firm clots in the blood collection tubes. These results demonstrate that rEcarin efficiently clots normal blood as well as blood spiked with high concentrations of anticoagulants and has great potential as an additive to blood collection tubes to produce high quality serum for analyte analysis in diagnostic medicine.

## 1. Introduction

Snake venoms contain relatively large amounts of prothrombin activators (PAs). These PAs can clot blood with high efficiency, without being subject to the regulation observed with human Factors X and V [1,2]. Snake prothrombin activator sources include those from *Oxyuranus scutellatus* (Australian Coastal Taipan (OsPA) that from *Pseudonaja textilis* (Australian common brown snake) (PtPA) and ecarin from *Echis carinatus* [3,4,5,6,7]. Recently, we employed OsPA for the rapid preparation of consistently high-quality serum using blood from both normal individuals and anticoagulated patients [5,6]. We demonstrated that OSPA converted prothrombin to thrombin in the blood sample, generating [5,6] a bolus of thrombin significantly greater than the amount used in commercially available thrombin tubes [5,6]. However, the use of snake prothrombin activators, such as OsPA and PtPA, in commercial blood collection tubes is problematic due to the supply considerations associated with procuring venom from snakes in quantity, quality and continuity.

Modern advances in genomics, proteomics and bioinformatics have driven the development of efficient biological expression systems [8,9]. These systems are widely employed to produce recombinant proteins for use in industrial and medical fields [8,9]. Both OsPA and PtPA form stable high molecular complexes (approximately 300 kD). They consist of heavy and light chains for both a Factor Xa-like protein and a Factor Va-like protein homologous with the human prothrombinase complex [10]. The complete cDNA sequence of a factor Xa-like protease from *P. textilis* has been determined and expressed in human kidney cells [11]. Although the molecule expressed from this cDNA sequence was capable of blood coagulation, it was produced in very low yields [11]. Although the Factor V-like protein from *P. textilis* escapes hemostatic regulation through procoagulant adaptions, a recombinant form expression has not been achieved [12]. In contrast, the prothrombin activator *ecarin* gene from the saw scaled viper (*Echis carinatus*) venom codes for a single chain polypeptide of 616 amino acids and is readily expressed in recombinant form [13]. It is a metalloproteinase with a molecular weight of 50–70 kDa dependent on the degree of glycosylation. This single chain metalloproteinase has an advantage over prothrombin activators in that it does not require other cofactors, Ca^2+^, or phospholipids [14]. As with other snake venom prothrombin activators, it is minimally or not affected by any regulatory components of the blood coagulation-fibrinolysis system in humans, e.g., Activated Protein C [5,6,15]. N-Ecarin has been used for measuring the total prothrombin in plasma. Ecarin clotting time (ECT) is a universal method used to monitor the activity of direct thrombin inhibitors, such as Hirudin, argatroban and dabigatran [16]. However, the use of ecarin purified from snake venoms has many of the drawbacks of OsPA and PtPA; therefore, it is desirable to have a recombinantly expressed form. The cloning and expression of ecarin in active form, including within the metalloproteinase domain, have been previously described in different systems [13,17,18,19]. However, it has not been demonstrated whether these recombinant forms can be scaled up to produce a commercial supply of recombinant ecarin. In addition, no study has reported the application of recombinant ecarin *in* medical practice.

Thrombin-containing blood collection tubes (RST) have been developed to speed up the clotting process when producing high quality serum for blood testing in a routine diagnostic laboratory setting, However the supplier does not recommend RST tubes for heparinized blood [20]. On the other hand, we have shown that snake venom OsPA, as an additive in blood collection tubes, produces an initial burst of thrombin and clots blood even in the presence of heparin [5,6]. OsPA also clotted heparinized bloods effectively to generate high quality serum with no effect on serum analytes [5,6]. As mentioned above, almost no regulatory components of the blood coagulation-fibrinolysis system in humans affects the activity of ecarin [19]. Ecarin also has no inhibitory effect on platelet aggregation [21]. Furthermore, ecarin has a high value of k_cat_ and a low value of K_m,_ indicating that the metalloproteinase is a highly specific and efficient enzyme [21]. Therefore, it is possible that rEcarin could be used as an additive in blood collection tubes to produce high quality serum.

In the present study, we expressed recombinant ecarin in mammalian cells followed by protein purification by standard chromatographic methods and characterization. We compared the clotting activity of rEcarin to that of N-Ecarin (Sigma Aldrich, St Louis, MO, USA) and demonstrated that it was effective as an additive in blood collection tubes when clotting normal blood, heparinized blood, and in the presence of other anticoagulants.

## 2. Methods

### 2.1. Cloning, Expression and Purification of Recombinant Ecarin (rEcarin)

Recombinant ecarin (rEcarin) is secreted as a propeptide of ~68–72 kDa (plus glycosylation), which is then cleaved to remove the pre-pro section. The resulting active ecarin molecule is 50–70 kDa (+glycosylation). The expression of the pro-protein is thought to be necessary for the correct folding of the protein and, hence, an active, mature form upon cleavage. An ecarin nucleotide sequence optimized for expression in mammalian cells was synthesized and cloned into the Invitrogen vector pcDNA 3.1. This sequence agreed with previously published data [13,18], with the addition of a TEV cut-site to facilitate activation. This construct was used to stably transfect HEK293 cells grown at 37 °C in an atmosphere of 5% CO_2_. rEcarin producing cells were detected in culture supernatant using the S-2238 (2 mM) chromogenic thrombin generation bioassay in a 96 well Nunc F plate at room temperature [22]. The highest consistent expression was gained from a HEK (Expi^TM^ 293) cell line [23]. A stable cell pool was generated by transfecting HEK cells with plasmid DNA containing the rEcarin gene sequence and selecting a stable cell pool using geneticin (200 µg/mL). This stable pool was used to manufacture batches up to 5 L using the bioreactor scale. Clones were isolated from the cell pool by limiting dilution, which produced a significantly higher ecarin yield/L of supernatant.

rEcarin was purified using a combination of three steps of column chromatography: Q Sepharose, Hydroxyapatite (HA), and CM Sepharose (Table 1). In short, the cell culture was harvested at 12–14 days, the supernatant isolated and then subjected to crossflow filtration where the supernatant was concentrated (10-fold) and buffer-exchanged into 50 mM HEPES, pH 7.4. An excess of TEV was added, and cleavage confirmed after 18 h by Western blot using an ecarin-specific polyclonal antibody (in-house source or commercial source) as well as a bioactivity assay (S2238). The digest was then purified by fractionation on IEX and affinity columns (unpublished data) before concentration and storage at −80 °C.

### 2.2. Western Blot

Purified rEcarin was separated by Nu-PAGE SDS-PAGE (4–12% gradient) and transferred onto a Hybond-P PVDF Membrane (Amersham, Buckinghamshire, UK). The PVDF membrane was blocked with 5% skim milk in PBST buffer (PBS with 0.1% tween 20) [24]. A primary polyclonal antibody against ecarin (in-house source or commercial source, 1:1000) was added to the membranes and incubated overnight at 4 °C with gentle rocking. On the second day, the membrane, after washing with 3× or 5 min with PBST, was incubated in secondary anti-Rabbit IgG (1:3500, Sigma #A0545) for 1 h with gentle rocking and then washed 3× for 5 min with PBST. The ecarin signal on the membrane was induced by using ECL Plus Western Blotting Detection Reagents (Thermo Fisher Scientific #32106, Waltham, MA, USA).

### 2.3. Analytical SEC Coupled with MALS of Purified rEcarin

The oligomeric profile of the final purified rEcarin was assessed using analytical Size-Exclusion Chromatography-high performance liquid chromatography (SEC). All analyzed samples were centrifuged immediately prior to analysis (20,000 g for 10 min at 4 °C) and the supernatant loaded into individual glass vials for analysis. The samples were kept at 10 °C until the experiment was carried out at room temperature. The experimental molecular weight of each species from the chromatography elution was assessed by Multi-Angle Light Scattering (MALS). The detailed parameters for the SEC-MALS data of the rEcarin were collected for analyzing the purity of rEcarin and its molecular weight (MW).

### 2.4. S-2238 One Stage MTP Ecarin Assay

Ecarin was assayed in a single reaction mixture containing prothrombin and the thrombin specific substrate S2238. The coupled reactions, prothrombin to thrombin and S2238 to pNA, monitored at 410 nm, result in non-linear progress curves of absorbance vs. time. These are analyzed by fitting second order polynomials to give the ecarin activity in milliUnits (mUs) defined as Nmol thrombin/min [22]. The S2238 assay was carried out to monitor the recovery of rEcarin from each column chromatography and the final yield (Table 1).

### 2.5. Clotting Assay of Whole Blood Samples in Both Commercial Blood Collection and rEcarin-Containing Tubes

Either fresh whole blood was added directly to tubes or for re-calcified, citrated blood, 50 µL of 1 M CaCl_2_ was added, followed by 3.95 mL of citrated normal whole blood (for each 4 mL total). Approximately 4–5 s of blood withdrawal was required before the clot began forming in the tube. The timer was initiated on the addition of the blood. The tubes were recapped immediately after the timer was started and these were gently tilted every 15 s for a total of 5 times at room temperature (RT). The clotting start times were estimated visually and recorded when clotting was first observed and also when a firm clot formed as defined by the clot staying in place upon inversion of the tube [6]. Several different commercial tubes were employed in the clotting experiments and these are indicated throughout the Results section. In the experiments, the rEcarin samples were dried in the tubes. Wet refers to material that was not dried and which was used for comparison in several experiments.

In the clinical trial, the clotting was timed and observed until completion at 5 min maximum following the addition of fresh whole blood into the rEcarin-containing tubes. The samples were then immediately centrifuged at 1300–1500× *g* (Centrifuge model: Eppendorf centrifuge 5810R, Hamburg, Germany) for 10 min at RT [25]. For the BDSST tubes, the clotting time was 30 min and these were centrifuged as per the recommendations at 1300–1500× *g* for 10 min. The rEcarin tubes were also centrifuged at 3000× *g* for 5 min. The serum was also examined visually for clarity, the presence of particulates, fibrin formation (rings, masses, or strands) and hemolysis. In some experiments, the serum was kept overnight to check for re-clotting.

### 2.6. Thromboelastography (TEG) of Citrated Whole Blood

The operating procedures for the TEG are provided in the TEG^®^ Haemostasis Analyser 5000 series (Haemscope Corporation, Niles, Illinois, USA) Operating Manual and accompanying software and as described elsewhere [26]. Approximately 15–20 s of blood withdrawal was required prior to clot initiation in the TEG cup. The TEG assay captures four important parameters (R time, K time, α-angle, and MA value). The R-value represents the time until the first evidence of clotting; the K value is the time from the end of R until the clot reaches 20 mm, representing the speed of clot formation; α-angle is the tangent of the curve made as K is reached; and MA is a reflection of clot strength [27]. Furthermore, a thrombus velocity curve (V curve) was calculated from the first derivative of changes in clot resistance to show a standard thromboelastographic tracing with three important thrombus generation parameters: (1) MRTG-maximum rate of thrombus generation; (2) TMRTG-time to maximum rate of thrombus generation; and (3) TTG-total thrombus generation. The details of the TEG assay were the same as described previously [5,6].

### 2.7. Statistical Analysis

Fisher’s exact test was used to analyze any difference in fresh whole blood clotting activities between N-Ecarin and rEcarin for two volunteers in two separate experiments with duplicates [28]. The *p* values reflect whether the two ecarins differ in their blood clotting activities (Excel-2013 (Formula-Statistical program) software was used for all analyses with Student’s two tailed *t* test and one way analysis of variance being employed. The patient and sample numbers, together with numerical values including mean ± standard deviation, are included in Figure 4. Power analysis was employed to determine the deference between N-Ecarin and rEcarin (Figure 2).

### 2.8. Human Research Ethics

The study was conducted in accordance with the Declaration of Helsinki. Human research ethics approval for this study involving blood collection from volunteers and patients was obtained from Metro South Human Research Ethics Committee and The University of Queensland Human Ethics Committee: HREC Reference number: HREC/08/QPAH/005. Most recent date of approval on 21 March 2017. The supply of human blood for research with ethics approval was obtained from the Australian Red Cross Service (ARCBS), Brisbane.

## 3. Results

### 3.1. Recombinant Ecarin (rEcarin) Expression, Purification and Identification

**Figure 1:**
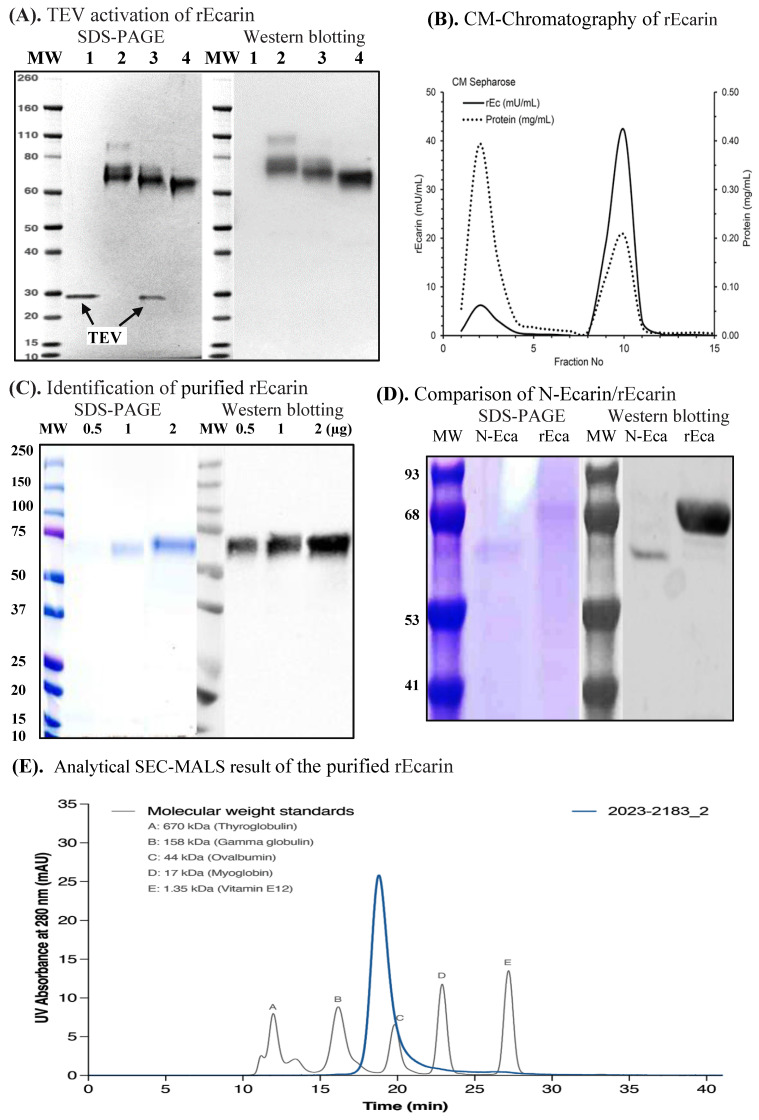
Activation, purification, identification of recombinant ecarin (rEcarin). (**A**) TEV activation of rEcarin analyzed by polyacrylamide gel electrophoresis (SDS-PAGE) staining (Left hand panel) and Western blotting assay by an anti-FLAG-HRP conjugate antibody (Origene) (Right hand panel). **1.** Tobacco etch virus protease (TEV) only, *Arrows* showing TEV in Lane 1 and 3. **2.** Full length of rEcarin. **3.** Full length of rEcarin with TEV activation. **4.** Full length of rEcarin with Aminophenyl mercuric acetate (APMA) activation; 2 µg of TEV only was loaded in Lane 1; 10 µg of unpurified rEcarin were loaded in Lane 2, and 4; 10 µg of unpurified rEcarin containing 2 µg of TEV were loaded in Lane 3. (**B**) CM-Chromatography of rEcarin. Combined fractions from the CHA column in low salt buffer were applied manually to a 20 mL column of CM-Sepharose, washed through with the same buffer, and eluted stepwise in 10 mL fractions with 0.5 M NaCl in the same buffer. rEcarin containing fractions were pooled (shaded), concentrated and buffer exchanged into 25 mM Hepes pH 7.4, 0.01% Tween 20. (**C**) Identification of purified rEcarin (1224-P9) by SDS-PAGE and Western blotting assay; 0.5, 1 and 2 µg of the purified rEcarin were loaded for each lane for SDS-PAGE staining (Left hand panel) and Western blotting assay (Right hand panel). (**D**) Comparison of N–Ecarin with rEcarin by SDS-PAGE and Western blotting assay; 2 µg of both N–Ecarin and purified rEcarin were loaded on one gel lane for SDS-PAGE staining (Left hand panel) and Western blotting (Right hand panel). An inhouse produced anti-ecarin polyclonal antibody, prepared in a rabbit, was employed for the Western blotting. (**E**). A representative SEC of the purified rEcarin.

Ecarin is expressed as a preproprotein generating an inactive N-glycosylated proprotein (68~72 kDa) that is activated by removal of the pro-peptide resulting in active N-glycosylated mature rEcarin (60~65 kDa). We inserted a TEV protease site between the pro-peptide and mature protein forms (Supplementary Figure S1) to achieve complete activation after harvest. Ecarin was partially activated in the cell culture prior to harvest, presumably due to endogenous enzymes, with a specific activation step required (i.e., TEV protease treatment) to ensure complete and consistent activation, which would be required for reproducible commercial manufacture (Figure 1A). Activation was also achieved using chemical cleavage with aminophenyl mercuric acetate (APMA). Following expression and activation of the rEcarin, the activation was confirmed initially by a size change in SDS-PAGE separation and immunoblotting (Figure 1A). Figure 1A shows rEcarin having a molecular size of ~70 kDa (50 kDa protein plus glycosylation), which gave rise to mature rEcarin after the TEV protease treatment. Activation was also successfully achieved using the enzyme trypsin (results not shown). Stable cell pools and clonal cell lines from HEK (Expi^TM^) transfected cells were generated as described in the Methods section. Supernatant was isolated and subjected to crossflow filtration, concentrating (10-fold) using buffer exchange. The rEcarin was purified according to protocols using a combination of three steps of column chromatography: Q Sepharose, Hydroxyapatite (HA) and *CM Sepharose* established in our laboratory (Table 1). rEcarin purification was monitored by S2238 bioactivity assay prior to combining the fractions (Table 1, Figure 1B). A typical profile of ecarin elution using CM-chromatography is shown. Ecarin activity was detected in two peaks with the majority appearing in peak 2. The specific activity of the high purity rEcarin produced was approximately 250–300 mU/mg protein (Table 1). Identification of the purified rEcarin appears in Figure 1C, confirming previous deglycosylastion studies that showed ecarin produced from HEK cells was present as a glycoprotein containing 18–20 kDa glycosylation (Supplementary Figure S2), similar to amounts when expression was carried out in other mammalian cell lines [13,18]. This compares to the N-Ecarin, which has a molecular size of ~60 kDa reflecting a lesser amount of glycosylation (Figure 1D). A FLAG sequence was included on the C-terminus in the early work (Supplementary Figure S2) to allow for the production of high purity ecarin for characterization studies that included Analytical Size Exclusion Chromatography (SEC), Protein Mass Finger printing, and N-Terminal sequencing (results not shown). The use of these techniques, together with SDS-PAGE separation and immunoblotting, confirm a close relationship of rEcarin with N-Ecarin.

Table 1 shows the purification and recovery of rEcarin from the three step column chromatography monitored by S2238 assay. A total of 400 mL of the supernatant crossflow from 4 L culture had 32,219 mU, approximately 8050 mU/L (Table 1). The recovery of rEcarin from the final CM Sepharose column was 76%, with a total of 24,403 mU, approximately 6100 mU/L (Table 1). Upon storage, the enzyme in pH 7.4 buffer with a stabilizing colloid was very stable over a period of at least one year as determined by the S2238 assay (results not shown).

We used an analytical SEC coupled with a MALS to assess the purity and molecular weight of the purified rEcarin. Results showed that purified rEcarin from different batches had over 90% purity, with one sample having the highest purity (97.57%) (Figure 1E), which also had a MW of 68 kDa, consistent with the WB results (Figure 1A,C,D). The results suggest that our established expression system and purification has the potential to facilitate large-scale production of rEcarin for medical applications.

### 3.2. Blood Clotting Characterization of rEcarin Compared with N-Ecarin

**Figure 2:**
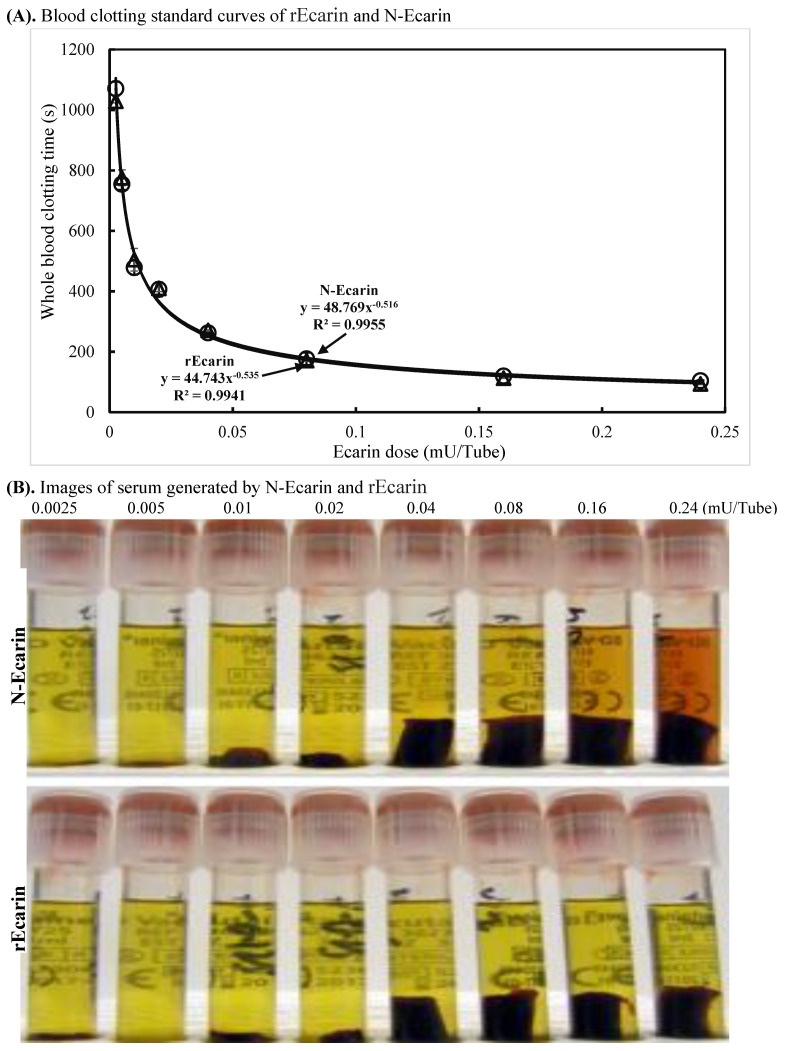

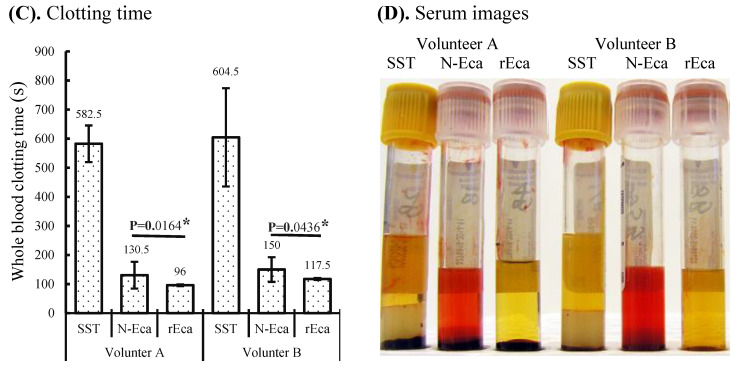
Characterization of rEcarin and N-Ecarin in clotting recalcified citrated whole blood and fresh whole blood. The activity of N-Ecarin and rEcarin in clotting both recalcified citrated whole blood and fresh whole blood. (**A**) Established standard curves of N-Ecarin and rEcarin when clotting 2 mL of recalcified citrated whole blood. (**B**) Image of serum generated by N-Ecarin and rEcarin showing that higher concentrations of N-Ecarin (0.16 and 0.24 mU) caused obvious hemolysis during blood clotting. The blood clots generated by 0.04, 0.08, 0.16 and 0.24 mU/tube showed clot retraction. (**C**) Activity of N-Ecarin and rEcarin (0.16 mU/Tube) when clotting fresh whole blood from two volunteers, compared with that of BD SST tube only, in duplicate (*n* = 2). Fisher’s exact test revealed that the clotting times generated by rEcarin were significantly shorter than those for N-Ecarin * *p* < 0.05. (**D**) Images of serum from the fresh whole blood of two volunteers clotted by BD SST tube, N-Ecarin- and rEcarin-containing red top plain tubes. N-Ecarin caused severe hemolysis during blood clotting.

Clotting performance for rEcarin was established in recalcified, citrated whole blood and compared with N-Ecarin, with the use of standard curves from low to high dose of the ecarin measured by the S2238 assay (Figure 2A). The standard curves in Figure 2A, which are identical for rEcarin and N-Ecarin, show clearly that ecarin at <0.05 mU/tube clots blood in less than 5 min. These curves are typical of the standard curves for snake venom prothrombin activators [6].

The plasma clotting times with increasing doses of both N-Ecarin and rEcarin showed a very similar pattern (Supplementary Figure S3). Clots produced by rEcarin were consistently rigid, giving rise to serum of a high clarity as determined visually (Figure 2B), lower panel). There was some hemolysis at higher concentrations of the N-Ecarin (0.16 and 0.24 mU/tube) which was not observed with rEcarin and may be caused by impurities from the venom used for the N-Ecarin (Figure 2B, upper panel). When fresh blood from two volunteers was tested, the clotting times generated by rEcarin were significantly shorter than those with N-Ecarin (Figure 2C). This could point to a tighter and perhaps stronger clot with rEcarin. Hemolysis was also evident when fresh blood was clotted using N-Ecarin and this did not occur with rEcarin (Figure 2D).

### 3.3. rEcarin Effectively Clots Anticoagulated Blood

As many as 10% of patients in a tertiary hospital setting are receiving anticoagulants [25,29]. These anticoagulants include heparin, clexane (low molecular weight heparins), fondaparinux (heparin analogue), warfarin, dabigatran (thrombin inhibitor) and rivaroxaban (Factor Xa inhibitor). Thus, having a blood collection tube additive that generates quality serum in the presence of anticoagulants is highly desirable. Therefore, we investigated the capacity of rEcarin to clot blood in the presence of a variety anticoagulants.

#### 3.3.1. Heparin

**Figure 3:**
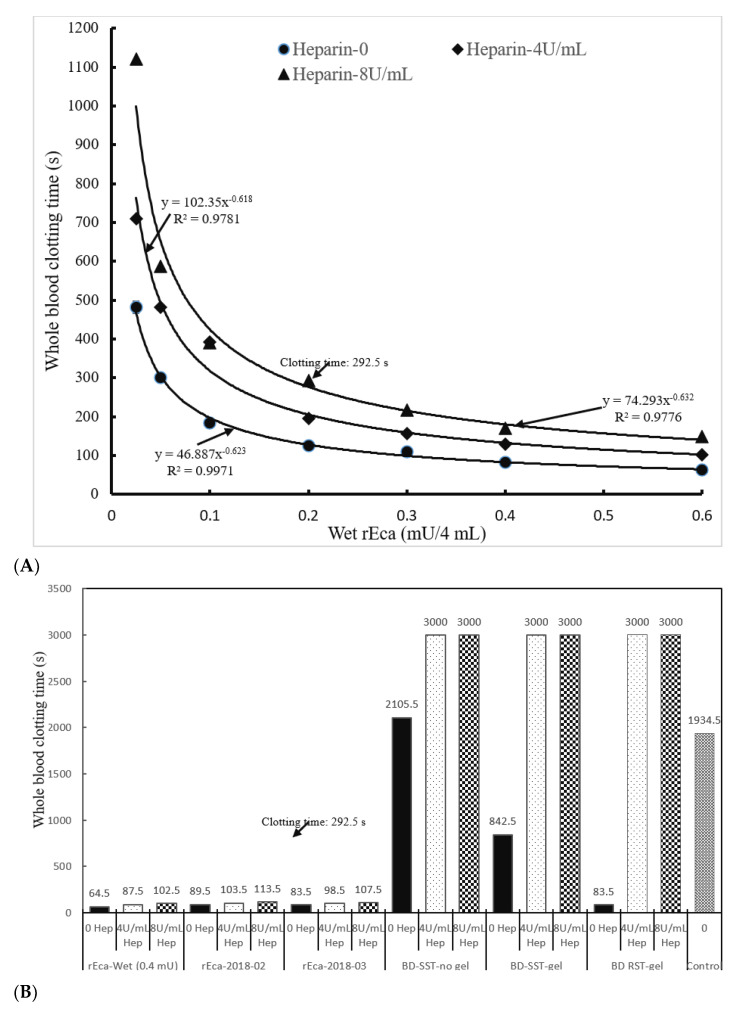
Potential of rEcarin as an additive in blood collection tubes for clotting heparinized blood. Both wet and dried rEcarins effectively clot recalcified citrated whole blood with or without heparin. (**A**) Standard curves of wet rEcarin in a GBO white top plain tube efficiently clots recalcified citrated whole blood spiked with two doses of heparin (4 and 8 U/mL heparin) and without heparin. (**B**) rEcarin (0.4 mU/tube) dried in a GBO white top plain tube efficiently clots the recalcified citrated whole blood spiked with two doses of heparin (4 and 8 U/mL heparin), compared with that of three commercial blood collection tubes (BDSST-no Gel, BDSST with gel, and BD RST tube).

Heparin is the standard hospital based anticoagulant for patients on dialysis (represented by 4 U/mL in our study in vitro and by 8 U/mL when including infrequently used high doses for cardiac patients) [30]. We therefore established a standard curve for the clotting of heparin-spiked blood samples (recalcified citrated whole blood). The results in Figure 3A show that efficient clotting occurs when heparin is present, and even at 8 U/mL of heparin, the clotting is complete in less than 5 min at 0.2 mU of rEcarin. This pattern was observed with separately purified rEcarin samples dried in GBO plain tubes (Figure 3B). In all cases, clotting was achieved in <2 min, even in the presence of 8U/mL heparin, and the process of drying had no effect on clotting times (Figure 3B). The results also reveal dramatic differences between the clotting times for rEcarin, and those in standard SST tubes with or without gel, and in RST tubes in the absence of rEcarin. No clotting was observed at 50 min (3000 s) in all types of commercial tubes (Figure 3B and Figure S4). The results further show that BDRST (thrombin) tubes did not clot the heparin-spiked blood over 3 U/mL whether it was spiked with Li-heparin or Na-heparin (Supplementary Figure S4). Although the BDRST tubes could clot 1 U/mL Li-heparin-spiked blood in 122.5 s, clotting with the 1 U/mL Na-heparin took was over 300 s. Furthermore, in 0.16 mU of N-Ecarin clotted heparinized blood (8 U/mL), hemolysis was again observed, and the clotting time was significantly longer (506 s) compared with the same number of activity units of rEcarin (291 s) (Supplementary Figure S5).

To confirm the visual blood clotting results, a TEG assay was conducted. Results showed that heparinized blood (8 U/mL) in a BDRST tube had an R time of 5700 s without any clotting and none of the other three clotting parameters (K time, angle-α and MA value) gave measurable data. V curve analysis supported that the heparinized blood in a BDRST tube had little generation of total thrombus (TTG) (8.27 mm), together with a very low maximum rate of thrombin generation (MRTG) (0.10 mm) and a prolonged time to maximal rate of thrombus generation (TMRTG) (16.58 min) (Supplementary Figure S6A). In contrast, the heparinized blood in a plain GBO tube containing wet rEcarin (0.20 mU/tube) had an R time of 90 s, together with a K time at 100 s, angle-α at 70.4, and MA at 76.3 mm, indicating very good clotting efficiency (Supplementary Figure S6B). The V curve analysis further revealed that the wet rEcarin-containing GBO tube generated an MRTG at 16.47 mm and a short TMRTG at 3.67 min and produced 913.07 mm of TTG for the heparinized blood at 8 U/mL (Supplementary Figure S6B). Furthermore, the heparinized blood in the plain GBO tube containing dried-rEcarin (0.2 mU/tube) showed that efficient clotting occurred, and with four good clotting parameters produced (R time at 110.0 s, K time at 150 s, angle-α at 57.2 and MA at 37.3 mm) and three thrombus generation parameters (MRTG at 9.43 mm/min, TMRTG at 3.57 min and TTG at 450.59 mm) (Supplementary Figure S6C). All the data supported the visual clotting analysis, confirming that rEcarin effectively clots heparinized blood at 8U/mL.

#### 3.3.2. Warfarin

**Figure 4:**
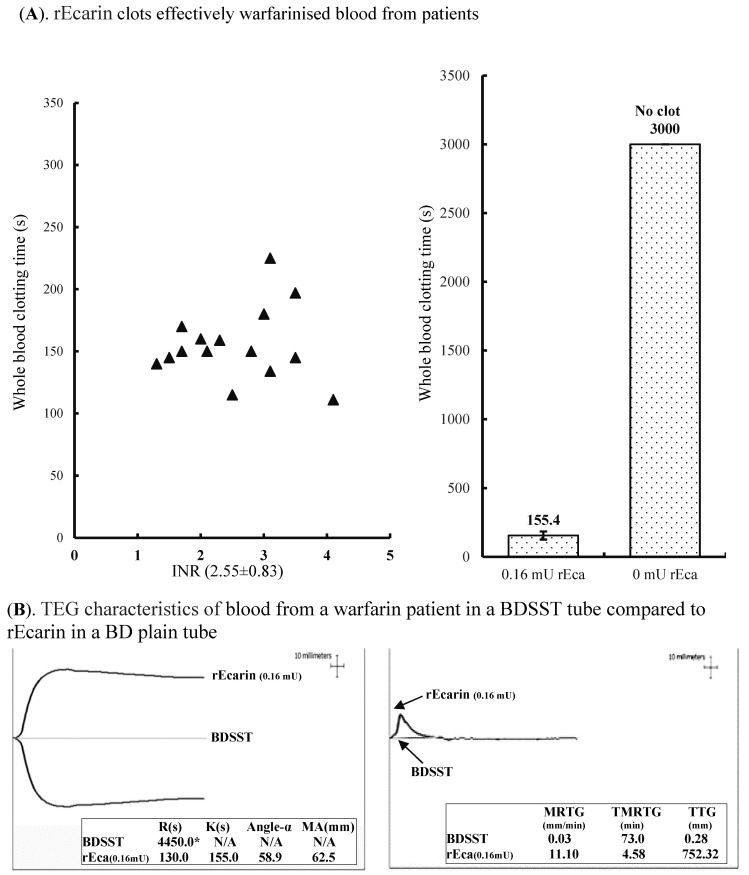
rEcarin effectively clots blood from patients on warfarin therapy. (**A**) rEcarin efficiently clots recalcified citrated whole blood samples from 15 patients being treated with warfarin at 1.3–4.2 of international normalized ratio (INR) (an average of INR at 2.55 ± 0.83). *Left panel*: rEcarin (0.16 mU/4 mL blood) effectively clotted the warfarinized bloods from 15 patients ranging from 112 to 227 s. *Right panel*: The average clotting time of blood from 15 patients by rEcarin (0.16 mU). The warfarinized blood did not clot at 3000 s without the addition of rEcarin. (**B**). The thromboelastography (TEG) assay shows TEG images of one warfarin patient’s blood in a BDSST tube and a BD plain tube containing 0.16 mU of rEcarin, revealing significantly different thromboelastographic traces of the same recalcified blood and four TEG parameters (R times, K time, angle-α and MA value) shown in inserted squares (*Left hand panel*), and the different V curves and three thrombin generation parameters (MRTG, TMRTG and TG) shown in the inserted squares (*Right hand panel*).

One unique property of snake venom prothrombin activators is that they activate descarboxyprothrombin (which is produced in the liver of warfarinized patients instead of prothrombin) [31]. Therefore, warfarinized blood should clot in a similar time to normal blood in the presence of rEcarin. For warfarin-containing whole blood, the clotting time for 15 patients (INR: 2.55 ± 0.83) ranged from 111 to 227 s in the presence of 0.16 mU rEcarin (Figure 4A, left hand panel). The average clotting time for these patients was 155 ± 29 s with rEcarin, while the warfarinized blood samples without rEcarin did not show any clotting up to 3000 s (Figure 4A, right hand panel). Furthermore, a TEG assay showed that whole blood from one warfarin patient in a BDSST tube did not show any clotting at 4450 s (R time), and no data for K time, angle-α or MA value were generated (Figure 4B, left hand panel). In contrast, the warfarinized blood in a BD non-additive plain tube containing 0.16 mU of rEcarin produced very good clotting, with an R time at 130 s, a K time at 155 s, angle-α at 58.9 and MA at 62.5 mm (Figure 4B, left hand panel). V curve analysis showed that the warfarin patient’s blood in a BDSST tube had a very low MRTG (0.03 mm), a prolonged time to TMRTG (73 min), and few TTG (0.28 mm), while the blood in a BD plain tube containing 0.16 mU of rEcarin had an MRTG at 11.10 mm, a very short TMRTG (4.58 min), and produced 752.32 mm of TTG (Figure 4B, right hand panel). The data clearly demonstrate that rEcarin works effectively in clotting blood from warfarinized patients, producing clotting times and TEG parameters similar to non-warfarinized blood.

#### 3.3.3. Dabigatran

**Figure 5:**
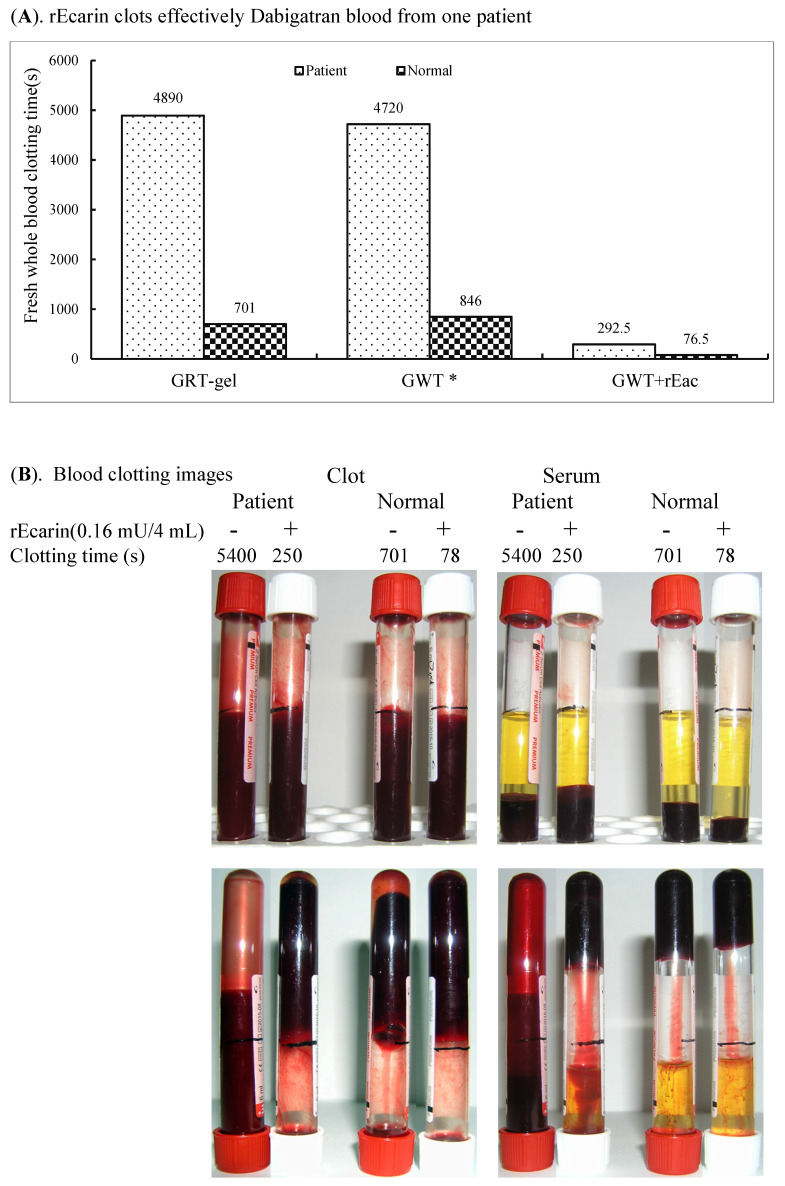
rEcarin clots effectively blood from one patient on dabigatran therapy. (**A**) Clotting time of the dabigatran-containing whole fresh blood sample from a patient volunteer who was treated with dabigatran with 0.16 mU rEcarin in 4 mL blood, compared with that of normal blood. Data are the mean±standard deviation (SD) of two time points (*n* = 4). GRT = Commercial GBO red top blood collection tube; GWT = Commercial GBO white top blood collection tube; GWT+ rEcarin = Commercial GBO white top blood collection tube + 0.16 mU rEcarin for 4 mL of blood in clotting assay. (**B**) Clotting images of one Dabigatran-containing blood sample from a patient volunteer who was treated with dabigatran with or without 0.16 mU rEcarin in 4 mL of blood, compared with that of normal blood at the first time point assay.

Since the thrombin antagonist dabigatran, which prevents blood clots and stroke in patients with atrial fibrillation, is a widely used anticoagulant [32], we tested the ability of rEcarin (0.16 mU/4 mL blood) to clot a patient’s blood who is prescribed dabigatron. The patient was on a dose of 100 mg of dabigatran with blood collected 5 h after the morning dose for a clotting assay twice a week (Figure 5A,B). With 0.16 mU of rEcarin, the blood from the patient formed a firm clot at 292.5 ± 60.1 s, whereas in its absence, only weak clotting was observed at 4890 ± 721.2 s (Figure 5A). When the clotting images were examined, the effectiveness of ecarin-induced clotting was also evident. (Figure 5B).

#### 3.3.4. Other Anticoagulants

**Figure 6:**
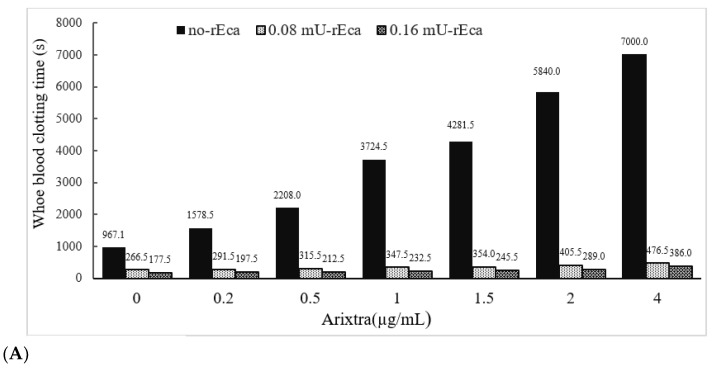

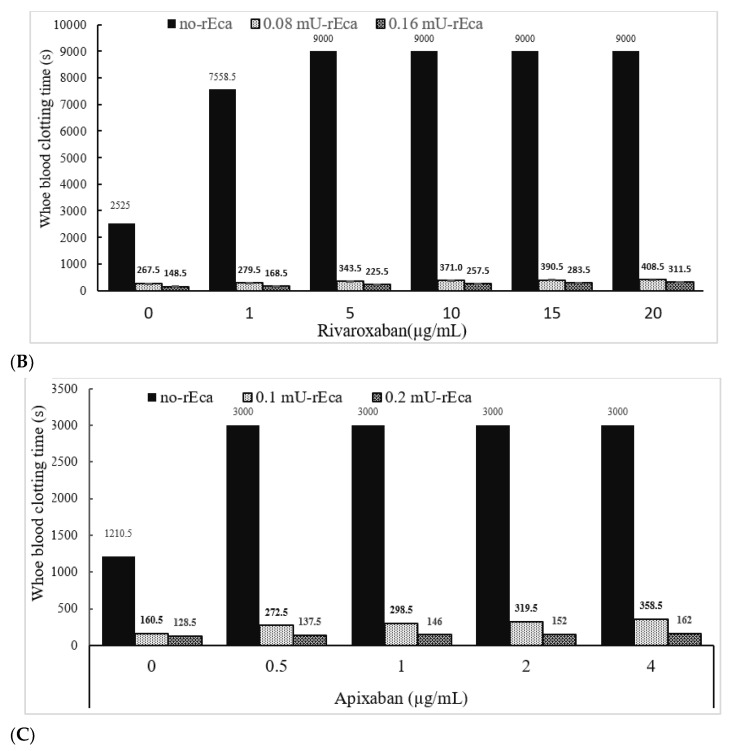
rEcarin effectively clots blood spiked with three anti-coagulants. The activity of rEcarin when clotting recalcified whole blood spiked with three different anti-coagulants (Arixtra, Rivaroxaban and Apixaban). (**A**) Time taken by rEcarin to clot recalcified whole blood spiked with Arixtra. Blood containing 4 µg/mL of Arixtra or more did not clot without the supplementation of rEcarin. (**B**) Time taken by rEcarin to clot recalcified whole blood spiked with rivaroxaban. Blood containing 0.5 µg/mL of Apixaban or more did not clot without the supplementation of rEcarin. (**C**). Time taken by RAPClot to clot recalcified whole blood spiked with Apixaban rivaroxaban. Blood containing 5 µg/mL of rivaroxaban or more did not clot without the supplementation of rEcarin. The data are the mean of duplicate assays.

We have also determined the activity of rEcarin in clotting recalcified citrated whole blood spiked with three other anticoagulation agents: fondaparinux, rivaroxaban and apixaban (Figure 6). Fondaparinux is used to prevent blood clots forming in patients who are recovering from orthopedic or abdominal surgery [33], while rivaroxaban functions to treat and prevent deep venous thrombosis (DVT) [34]. In addition, apixaban enables blood to flow through a patient’s veins more easily to prevent dangerous blood clot formation [35]. In these experiments, both Fondaparinux and apixaban were spiked into citrated blood, with a range of 0–4 µg/mL, and rivaroxaban with a range of 0–20 µg/mL (Figure 6). All doses used for the three anticoagulants were significantly above those used clinically. As shown in Figure 6, the citrated blood spiked with the three anticoagulants showed significant prolongation of clotting times or failure to clot. Clotting images showed no blood clotting occurred in apixaban-containing blood (0.5 µg/mL) (Supplementary Figure S7A,B). Centrifugation led the apixaban contained blood at 0.5 µg/mL to generate latent clotting, or reclotting, while the blood containing over 1 µg/mL did not generate any latent clotting and only plasma was produced (Supplementary Figure S7B, lower panel). In contrast, in the presence of 0.16 or 0.2 mU rEcarin in a tube with 4 mL blood, effective clotting occurred even at the highest doses of all three anticoagulants after approximately 300 s (Figure 6A–C). All of these results demonstrate that rEcarin functions effectively to clot blood from both normal patients and those on anticoagulants to produce high quality serum.

## 4. Discussion

In this study, we investigated the expression of rEcarin in HEK cells using a combination of three steps of column chromatography for rEcarin purification. The result showed that a novel and scalable rEcarin can be developed to industry standards. We then determined the clotting performance of rEcarin in recalcified citrated whole blood, plasma, and fresh whole blood compared with N-Ecarin, confirming the important advantage of using rEcarin to produce high quality serum by preventing hemolysis. The rEcarin was shown to effectively clot not only the recalcified citrated or fresh whole blood, but also different anticoagulating bloods, forming firm clots in the blood collection tubes, and revealing a potential application of rEcarin in blood diagnostics. Although other agents that increase the clotting efficiency of blood have been reported and applied as hemostatic agents [36,37], they have not been used in the context described here.

The snake venom prothrombin activator ecarin is a metalloproteinase, synthesized as a single polypeptide chain and is subject to a lesser amount of post-translational modification than other snake prothrombin activators such as OsPA and PtPA [18,21]. Considerable energy input is required by the snake to produce ecarin-containing venom and it may take days or even several weeks to replenish stores of depleted venom [38]. Because of the difficulty in obtaining a readily available source of the snake venom, in addition to problems associated with its use for commercialization, we cloned and expressed a recombinant form of ecarin from the saw scaled viper (*Echis carinatus*) in a number of different cell lines including HEK cells. We observed that expression of rEcarin in a suspension of HEK cells (Expi^TM^) provided the highest yield of protein and total activity (see Table 1).

Previous results have reported the cloning and expression of active forms of rEcarin in both *E. coli* and CHO cells [13,18]. They revealed that ecarin was autoactivated with time in a culture medium. We also observed partial activation of ecarin in cell culture prior to harvest due to endogenous enzymes or autoactivation and, accordingly, introduced a TEV site into the construct, to ensure complete and consistent activation, which would be required for reproducible commercial manufacture. We also observed activation at a nearby site using trypsin (results not shown). Jonebring and colleagues reported a higher molecular weight form of the mature recombinant ecarin produced in CHO cells (*70 kDa) compared to the *E. coli* produced ecarin (*50 kDa), suggesting that the CHO-produced ecarin was glycosylated [13]. Similar to the CHO-produced ecarin, our HEK-produced ecarin had a molecular weight of ≈70 kDa; the glycosylation of which was determined by PNG-ase studies.

In *E. coli* expression systems, it has been estimated that modest protein expression of recombinant protein is between 50 and 150 mg/L (2–5% of the total cellular protein) and soluble proteins can be recovered with good yields (>50%) [39]. Recently, transient fibrinogen expression has been shown to rapidly generate yields of 8–12 mg/L fibrinogen in HEK (Expi293^TM^) suspension cells, with purification recovery of 50–75% of the recombinant fibrinogen [40]. According to the Jonebring et al. study, the highest yield of CHO- produced ecarin was up to 7000 EU ecarin/litre in lab scale shaker cultures (equivalent to ~5500 mU/L), but the recovery percentage of the purified rEcarin was not reported [13]. Very recently, Mohammadi et al. reported that the ecarin metalloproteinase domain was expressed in *E. coli*, with high plasma clotting activity, but it is unclear whether expression of the metalloproteinase domain could be scaled up to produce high yield [17]. In our study, we obtained a purification recovery of 76% of rEcrain and the yield of the purified rEcarin was 18.7 mg/L, with the prothrombin activity at 6100 mU/L (7820 IU/L). Further batches have been produced with higher yields, with 25–50 mg/L in shaker flasks suggesting that we have initially established an expression system that has the potential for scalability in clonal cell lines.

In the present study, rEcarin rapidly clotted both recalcified citrated whole blood and fresh whole blood containing different anticoagulants including heparin, warfarin, dabigatran, fondaparinux, apixaban and rivaroxaban, forming stable clots in vitro. In the clinic, heparinized bloods from anticoagulated patients usually contain concentrations in the range of 1–8 U of heparin/mL, which prevents clotting [41,42]. Here, we found that 0.2 mU rEcarin efficiently clotted 4 mL of heparinized blood at 8 U/mL in less than 300 s, producing a fully formed clot. This is consistent with our previous observation that the Australian snake venom prothrombin activators, PtPA and OsPA, formed a solid blood clot for heparinized blood at the same high concentrations of heparin [5]. Furthermore, OsPA, which is homologous to the mammalian prothrombinase complex, rapidly promoted clotting of whole blood and is capable of activating normal prothrombin and also descarboxy-prothrombin without the need for cofactors [5,6], with similar clotting times between non-anticoagulated and warfarinized patients [5]. Consistent with our previous results using OsPA, rEcarin was shown here to effectively clot blood from patients on warfarin. The average clotting time for 15 patients on warfarin (INR: 2.15 ± 0.83) was approximately 2.5 min in the presence of rEcarin (0.16 mU), while in its absence, no clotting was observed up to 50 min. The quality of the clots was also excellent when determined by TEG parameters.

Although ECT is unaffected by prior treatment with warfarin or the presence of phospholipid-dependent anticoagulants, such as lupus anticoagulant [43], ecarin based assays were rarely used for anticoagulation monitoring of the direct oral thrombin inhibitor dabigatran etexilate in 2010 [44]. Here, blood from a patient on dabigatran therapy only showed a soft clot after 80 min in a conventional blood collection tube; however, the presence of 0.15 mU rEcarin caused a firm clot in less than 5 min to produce high-quality serum. In addition to heparin, warfarin and dabigatran, a range of other anticoagulants, such as fondaparinux, apixaban and rivaroxaban, are used clinically [16,45]. Here, we further demonstrated the efficacy of rEcarin in clotting recalcified citrated whole blood spiked with these three anticoagulants at doses significantly higher than clinically used levels.

The capacity of rEcarin to rapidly promote blood clotting in the presence of high levels of anticoagulants, both in spiked samples and in patients, represents an important advance in improving serum quality for reliable analyte determination. The significance of this is evident from the data that reveal as many as 10% of patients in a tertiary hospital setting are receiving anticoagulants [25,46]. The total sales for the top four oral anticoagulants for 2015 was estimated at $US 7.8B [47]. It is noteworthy that while demand for these anticoagulants is increasing, there is a decline in the more traditional anticoagulants such as warfarin. Thus, having a blood collection tube additive that ensures the generation of quality serum with newer anticoagulants is an increasing requirement. We have previously used the snake venom prothrombin activator, OsPA as an additive in blood collection tubes to rapidly produce high-quality serum suitable for analyte testing [5,6]. Thus, rEcarin as a snake venom prothrombin activator represents a promising candidate, not only as a procoagulant in blood collection tubes, but also might replace N-Ecarin for the ecarin clotting time (ECT) test.

The advantages of rEcarin are that it significantly decreases blood clotting time (<5 min) and clot strength is enhanced. The visual clotting time in the presence of rEcarin (0.16 mU/tube) was approximately 2 min, significantly lower than that in a plain tube (approximately 10 min). Determination of R times by TEG under the same conditions confirmed a 5-fold difference in clotting times. The maximum rate of thrombin generation (MRTG) increased markedly in a BD plain tube containing rEcarin as shown in Figure 4 and the total thrombin generation (TTG) was approximately 3000 times higher than in its absence. Here, the rEcarin produced meizothrombin to promote thrombin which converts fibrinogen to fibrin, resulting in a blood clot. However, the mechanism of production is different in that rEcarin activates human prothrombin into meizothrombin through a specific proteolytic cleavage, which then cleaves fibrinogen to enhance clot formation. In tubes to which rEcarin is added, there is a rapid and sustained generation of large amounts of meizothrombin. In addition, thrombin produced by this mechanism in snake venom PA tubes is not subject to anti-thrombin III inhibition. We have previously shown that this is also the case for other snake prothrombin activators [6].

Decreased clotting time in the presence of rEcarin is an important advantage in blood collection since attention has been drawn to the unreliability of sample collection and processing, which may impact on the stability of analytes [48,49]. For example, accurate registration of collection time is necessary for determining whether some tests should be performed or not (e.g., glucose, hormones, drug monitoring) [50]. We have shown that the quality of serum and analyte determination are unaffected by the inclusion of the snake venom prothrombin activator, OSPA [5,6]. Preliminary studies show that the use of rEcarin as a procoagulant in blood collection tubes with normal and heparinized blood samples did not adversely affect the serum analyte determinations (unpublished results). Further experiments are required to confirm these data.

## 5. Conclusions

In conclusion, a codon-optimized ecarin gene was cloned and expressed in a HEK cell line. The resulting rEcarin exhibited a high prothrombinase activity in clotting recalcified citrated whole blood, plasma, and fresh whole blood, comparable to native ecarin, without causing hemolysis. rEcarin also rapidly clotted both recalcified citrated whole blood and fresh whole blood containing different anticoagulants including heparin, warfarin, dabigatran, fondaparinux, apixaban and rivaroxaban, forming stable clots in vitro and producing high quality serum. Therefore, rEcarin can be used as an additive in blood collection tubes to enhance blood clotting and to produce high quality serum in a diagnostic setting and has the potential to be scaled up for commercial use.

## Figures and Tables

**Table 1:** Chromatographic Purification of rEcarin.

Fraction	Volume	Protein	rEc Concentration	rEc	Specific Activity	Purification	Recovery
(Concentrates)	(mL)	(mg)	(mU/mL)	(mU)	(mU/mg)	(fold)	(%)
Cross Flow	400	1687	80.5	32,219	19.1	1.0	100
Q Sepharose	252	703	131	32,927	46.9	2.5	102
Hydroxyapatite	32	239	845	27,040	113	5.9	84
CM Sepharose	150	75	163	24,403	323	17	76

Note: 400 mL of the supernatant crossflow was concentrated from 4 L of culture medium (10-fold).

## Data Availability

All data relevant to the manuscript are included.

## Supplementary Materials

The following supporting information can be downloaded at: https://www.mdpi.com/article/10.3390/biom12111704/s1, Figure S1: Deduced amino acid sequence of recombinant ecarin (rEcarin); Figure S2: Recombinant ecarin (Batch 2015-1224) denaturing PNGase F deglycosylation analysed by (A) SDSPAGE and (B) anti-FLAG western blot; Figure S3: Activity of N-Ecarin and rEcarin in clotting recalcified citrated plasma. Plasma samples were freshly prepared for clotting assay at room temperature; Figure S4: BDRST tube did not clot recalcified citrated whole blood spiked with either Li-heparin or Na-heparin over 3 U/mL; Figure S5: Activity of rEcarin in clotting recalcified citrated whole blood containing four doses of heparin (0, 4, 8 and 20 U/mL). Native ecarin at 0.16 mU/4 mL was used to clot heparinised blood at 8 U/mLof heparin; Figure S6: Thromboelastography (TEG) assay shows TEG images of recalcified citrated whole blood spiked with or without heparin at 8 U/mL in BDRST tube only and GBO plain tube containing either wet or dried rEcarin; Figure S7: Clotting images of GBO plain tube with or without 0.20 mU rEcarin in clotting 4 mL of blood sample spiked with 0, 0.5, 1.0, 2.0, and 4.0 µg of Apixaban/per m blood, respectively.
