# Supplementary material for: Potential Application of Recombinant Snake Prothrombin Activator Ecarin in Blood Diagnostics
Source: Biomolecules. 2022 Nov 17;12(11):1704. doi: 10.3390/biom12111704 (PMC9687618; doi:10.3390/biom12111704)

Figure S1: Deduced amino acid sequence of recombinant ecarin (rEcarin). (A). The deduced amino acid sequence includes signal peptide (red color), propeptide (blue color), TEV signal (black color) and mature ecarin (green color). (B). Diagram of rEcarin.

A. Signal peptide, Propeptide, Optional specific cleavage site (e.g.) TEV, Mature ecarin

MIQILLVIICLAVFPYQGCSIILGSGNVNDYEVVYPQKVTALPKGAVQQPEQKYEDAMQYEF
EVKGEPVVLHLEKNKELFSEDYSETHYSSDDREITTNPSVEDHCYYHGRIQNDAESTASISA
CNGLKGHFKLRGETYFIEPLKIPDSEAHAVYKYENIENEDEAPKMCGVTQDNWESDEPIKKT
LGLIENLYFQSPHERKFEKKFIELVVVVDHSMVTKYNNDSTAIRTWIYEMLNTVNEIYLPF
NIRVALVGLEFWCNGDLINVTSTADDTLHSFGEWRASDLLNRKRHDHAQLLTNVTLDHSTLG
ITFVYGMCKSDRSVELILDYSNITFNMAYIIAHEMGHSLGMLHDTKFCTCGAKPCIMFGKES
IPPPKEFSSCSYDQYNKYLLKYNPKCILDPPLRKDIASPAVCGNEIWEEGEECDCGSPADCR
NPCCDAATCKLKPGAECGNGECCDKCKIRKAGTECRPARDDCDVAEHCTGQSAECPRNEFQR
NGQPCLNNSGYCYNGDCPIMLNQCIALFSPSATVAQDSCFQRNLQGSYYGYCTKEIGYYGKR
FPCAPQDVKCGRLYCLDNSFKKNMRCKNDYSYADENKGIVEPGTKCEDGKVCINRKCVDVNT
AY

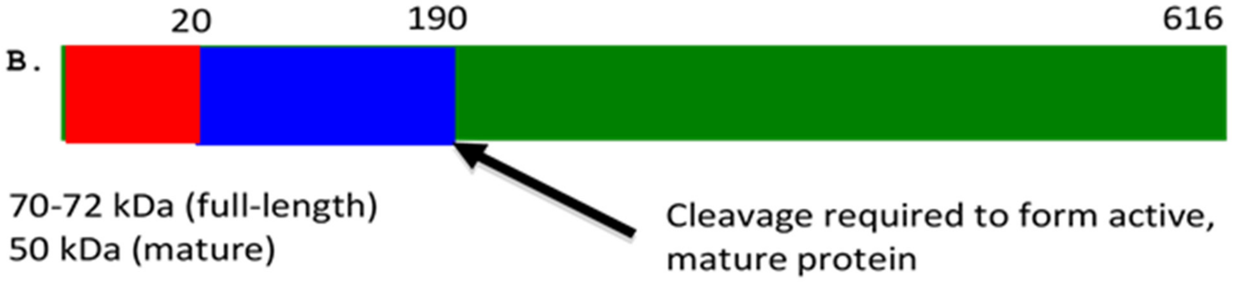

Figure S2: Recombinant ecarin (Batch 2015-1224) denaturing PNGase F deglycosylation analysed by **(A)** SDSPAGE and **(B)** anti-FLAG western blot. Sample order: **1).** Novex® Sharp Pre-stained MW ladder, **2)**. Full-length 2015-1224 (5x dilution), **3).** Full-length 2015-1224 (Deglycosylated) (2.5x dilution), **4)**. Cleaved 2015-1224(5x dilution) and **5)**. Cleaved 2015-1224 (Deglycosylated) (2.5x dilution).

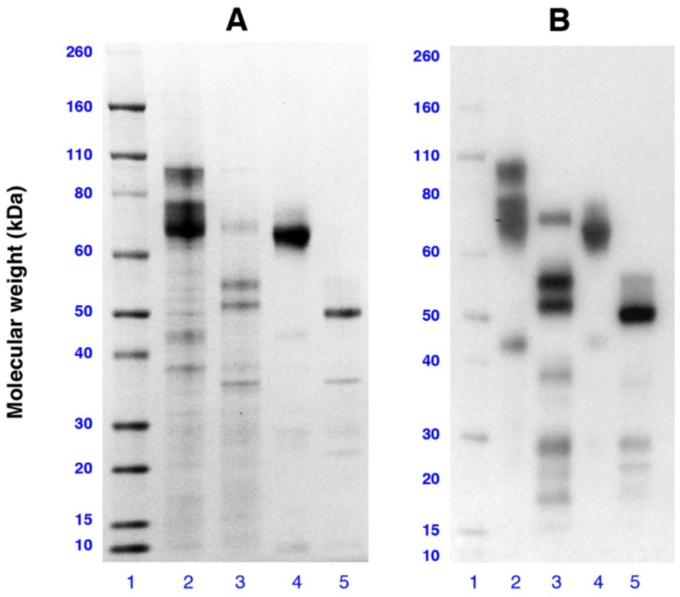

COMMENTS:

• Three major bands present in the full-length 2015-1224 sample shifted from approximately 70, 75 and 90-100 kDa to approximately 50, 55 and 70 kDa following PNGase F treatment. This represents a size difference of 20 kDa that can be attributed to N-glycosylation. It is unclear whether the 70 kDa band in the PNGase F treated sample represents the deglycosylated 90-100 kDa band or incompletely deglycosylated 70 kDa full-length protein.

• The major band present in the cleaved 2015-1224 sample shifted from approximately 65 kDa to 50 kDa upon treatment with PNGase F, representing a size difference of 15 kDa that can be attributed to N-glycosylation. This 50 kDa band appears to be somewhat smaller than the lowermost band of the PNGase treated full-length protein.

• Taken together, the results suggest that N-glycans constitute 15-20 kDa of the secreted 2015-1224 molecular weight. It also provides support for the hypothesis that the three full-length 2015-1224 bands represent different N-terminal truncated/degraded products.

Figure S3: Activity of N-Ecarin and rEcarin in clotting recalcified citrated plasma. Plasma samples were freshly prepared for clotting assay at room temperature. The recalcified citrated plasma clotting assay was performed using a Hyland-Clotek instrument as previously described (Zhao et al. *Clin Chem Lab Med* 2019, **57**(4):483-497). Freshly pooled citrated plasma from normal volunteers was used for each group of experiments. The assay volume was 250 µL. Citrated normal human plasma (100 µL) was added to a glass clotting tube (500 µL) with 100 µL of 0.05 M Hepes buffer with added 0.1 M NaCl (pH 7.4) and placed in the 37 °C heating block of a Hyland-Clotek plasma clotting machine (Hyland Division, Travenol Laboratories, Inc., USA). After at least 1 min, 25 µL of 0.2 M CaCl2 (to a final concentration 20 mM) was added (when required), immediately followed by the addition of 25 µL of a N-ecarin or rEcarin solution at different concentrations, at which point the timer was started. The data are the mean of duplicate clotting assays.

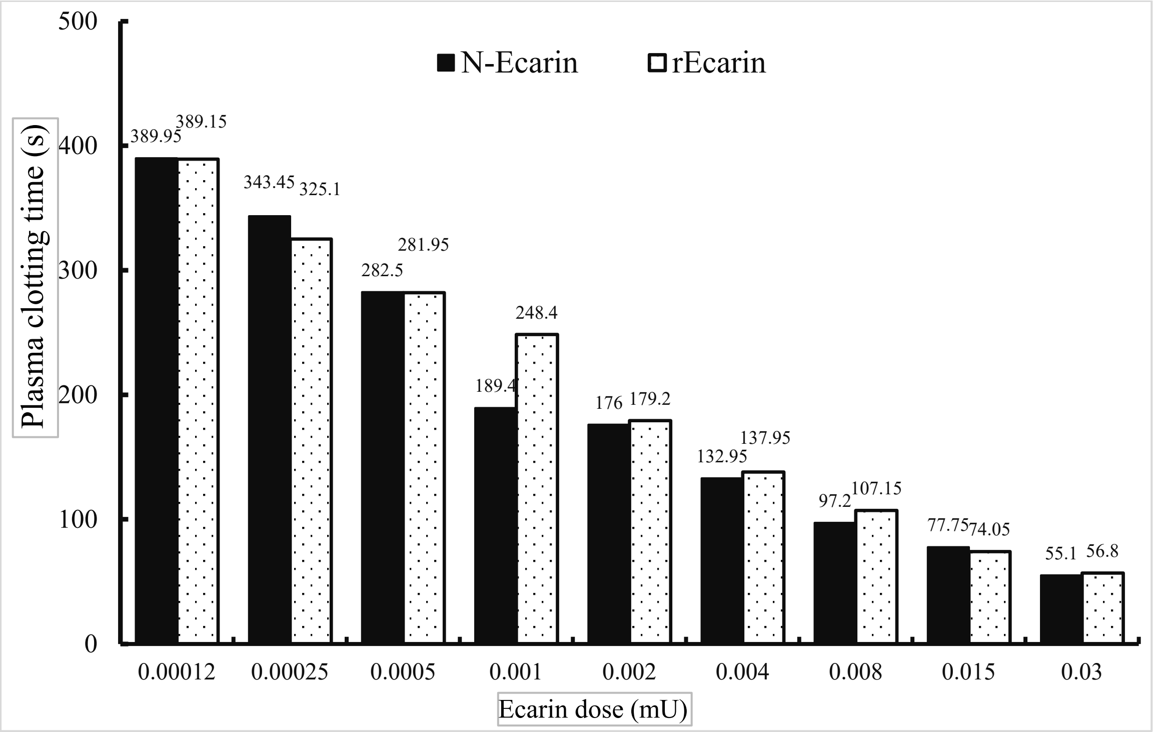

Figure S4: BDRST tube did not clot recalcified citrated whole blood spiked with either Li-heparin or Na-heparin over 3 U/mL.

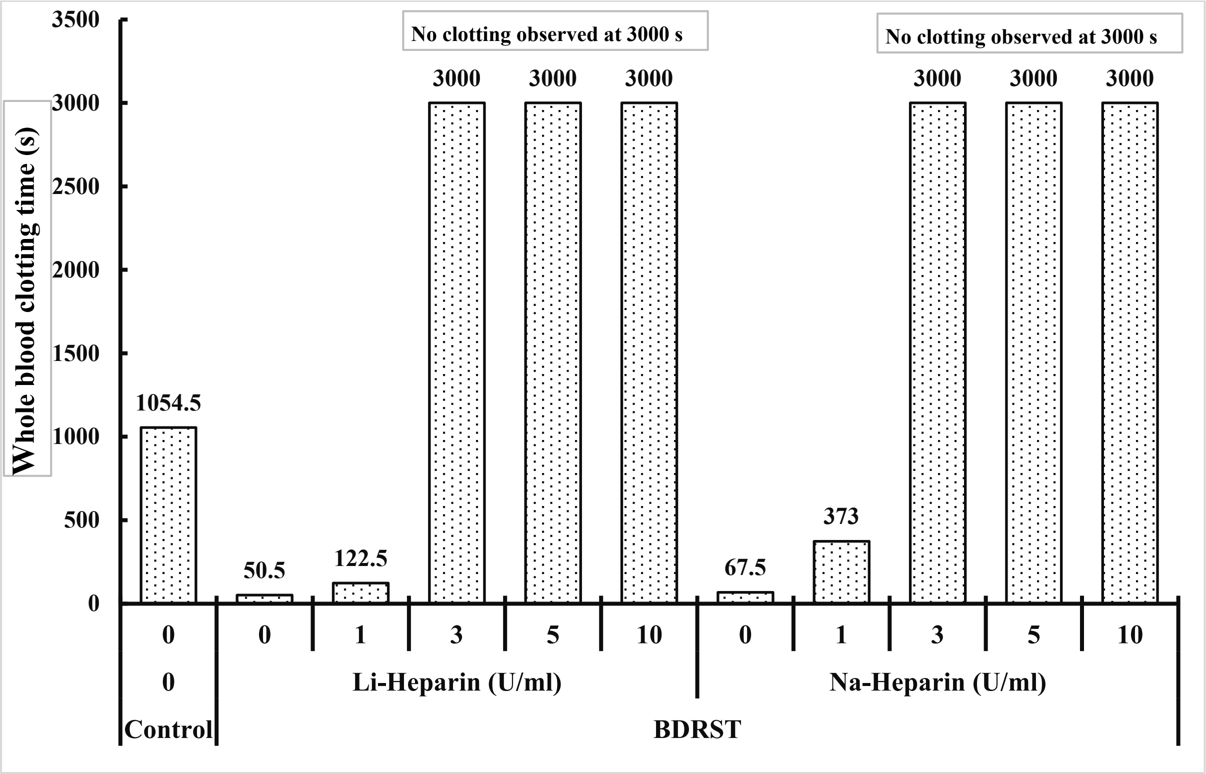

Figure S5: Activity of rEcarin in clotting recalcified citrated whole blood containing four doses of heparin (0, 4, 8 and 20 U/mL). Native ecarin at 0.16 mU/4 mL was used to clot heparinised blood at 8 U/mLof heparin.

Ecarin (mU/4 mL)	0	0	0	0.16	0.32	0.32	0.64	0.64	0.16* (N-Eca)
Heparin(U/mL)	0	4	8	8	20	8	20	8	8

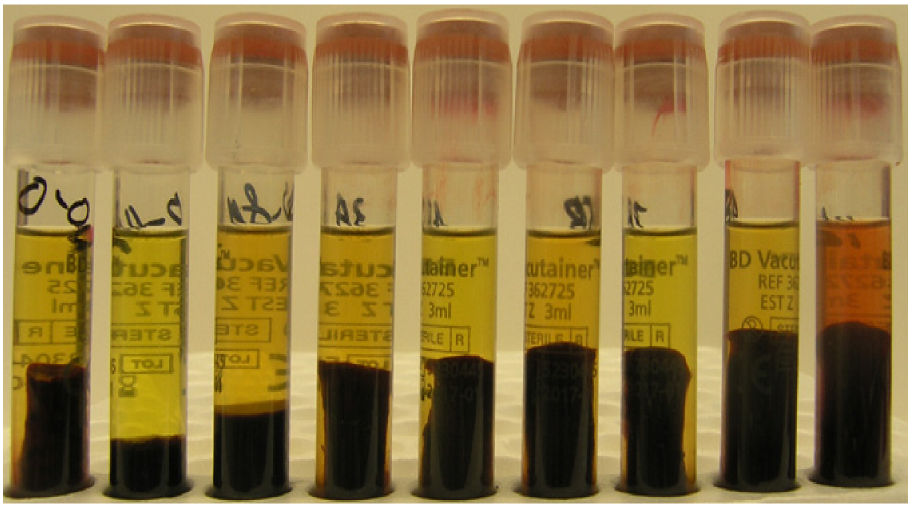

Clotting time (Sec)	1205	no clot	no clot	291.5	300	166	129.5	73.5	506.5

Figure S6: Thromboelastography (TEG) assay shows TEG images of recalcified citrated whole blood spiked with or without heparin at 8 U/ml in BDRST tube only and GBO plain tube containing either wet or dried rEcarin. **(A).** TEG assay for BDRST tube in clotting recalcified only or heparinised citrated whole blood. **(B).** TEG assay for GBO plain tube containing 0.2 mU wet rEcarin in clotting recalcified only or heparinised citrated whole blood. **(C).** TEG assay for GBO plain tube containing 0.2 mU dried rEcarin in clotting recalcified only or heparinised citrated whole blood. Four TEG clotting parameters (R times, K time, angle-α and MA value) showing in inserted squares (*Left hand panel*) and V-curves showing three thrombin generation parameters (MRTG, TMRTG and TG) showing in inserted rectangle boxes (*Right hand panel*).

A. BDRST/TEG assay

TEG

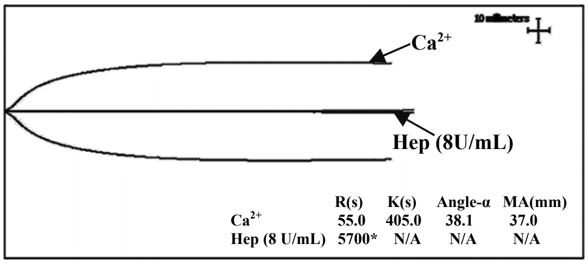

V-curve

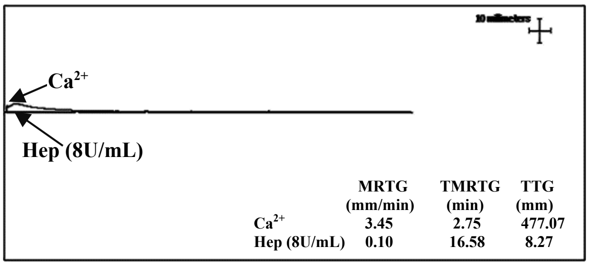

B. Wet rEcarin/TEG assay

TEG

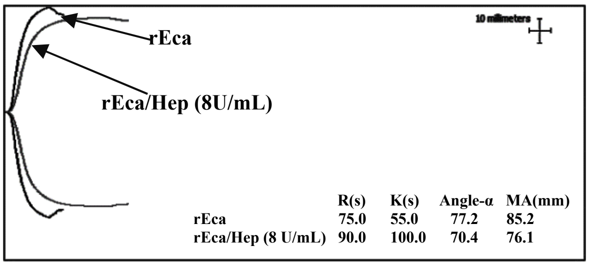

V-curve

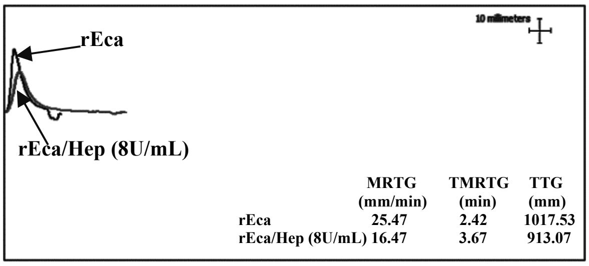

C. Dried rEcarin/TEG assay

TEG

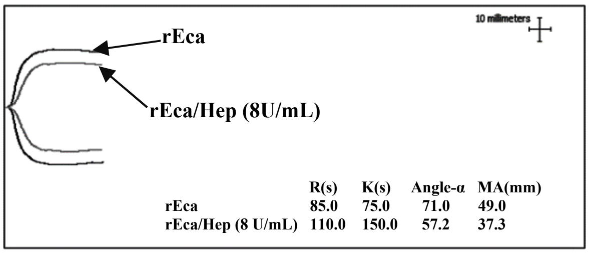

V-curve

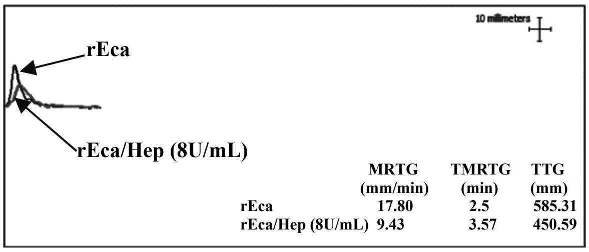

Figure S7: Clotting images of GBO plain tube with or without 0.20mU rEcarin in clotting 4 mL of blood sample spiked with 0, 0.5, 1.0, 2.0, and 4.0 µg of Apixaban/per m blood, respectively. **(A).** Clotting image was taken from the Apixaban-spiked blood without supplementary of rEcarin at 3000 s. **(b).** Serum/plasma image was taken from centrifugation of the Apixaban-spiked blood without rEcarin at 3000 s post-spike at 1300g for 10 min.

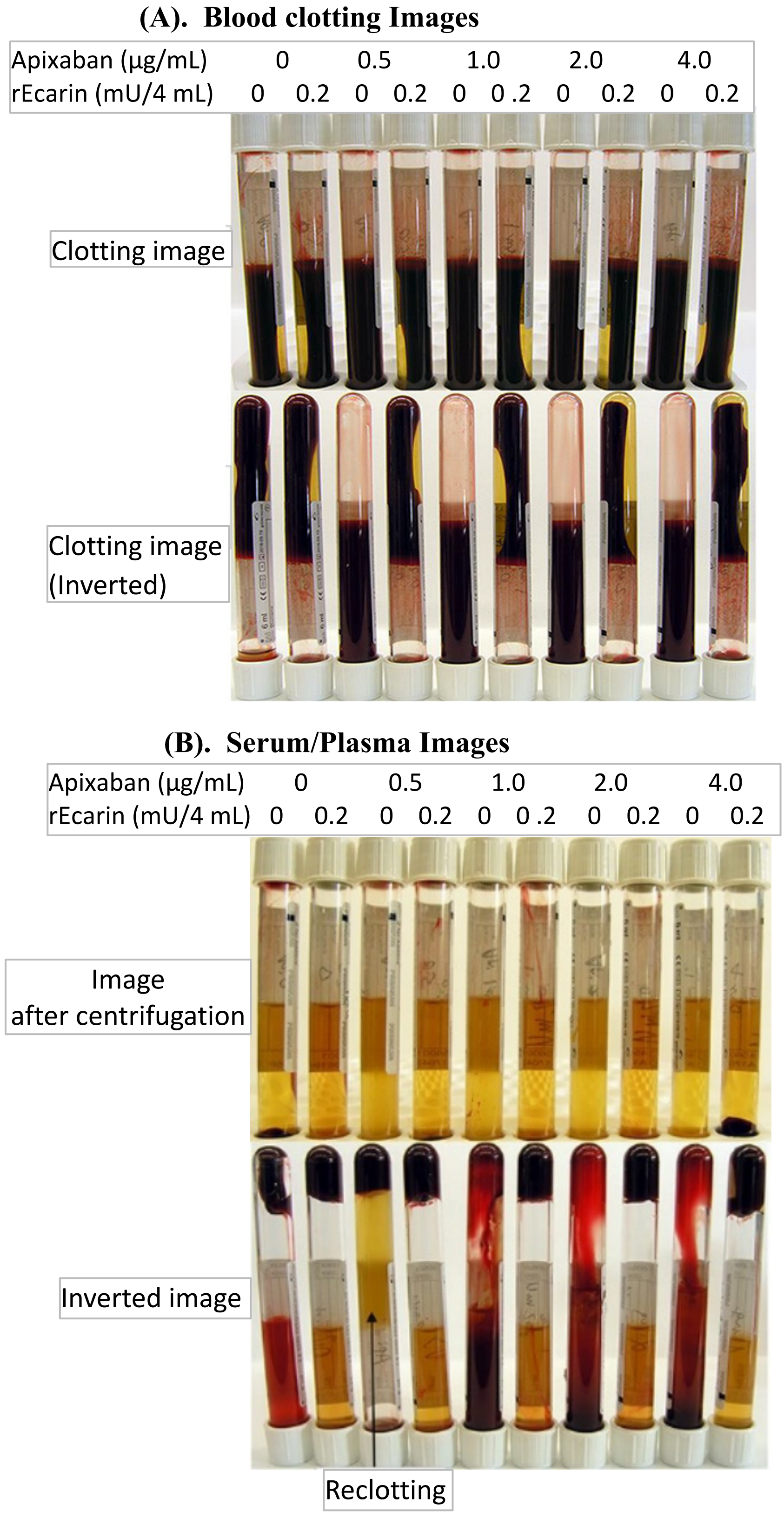

Supplement: Supplementary file 1 [file biomolecules-12-01704-s001.zip › biomolecules-1981798-supplementary.pdf]
